# Pseudoaneurysm rupture causing hemoperitoneum following rectal impalement injury: A case report

**DOI:** 10.1016/j.ijscr.2019.01.002

**Published:** 2019-01-19

**Authors:** Pyong Wha Choi

**Affiliations:** Department of Surgery, Ilsan Paik Hospital, Inje University College of Medicine, 170, Juhwa-ro, Ilsanseo-gu, Goyang-si, Gyeonggi-do, 10380, Republic of Korea

**Keywords:** Pseudoaneurysm, Rectum, Impalement injury, Hemoperitoneum

## Abstract

**Introduction:**

Although vascular anatomy of the rectum is complex, pseudoaneurysm followed by massive hemoperitoneum after rectal impalement injury is extremely rare.

**Case presentation:**

A 43-year-old man presented with abdominal distension. One day earlier, he had undergone sigmoid loop colostomy for rectal implement injury at a local hospital. After the operation, he had become hemodynamically unstable. Digital rectal examination showed a penny-sized anterior rectal wall defect 6 cm from the anal verge. Computed tomography (CT) revealed a hematoma (12 × 10 × 15 cm) with bleeding in the pelvic cavity and an adjacent pseudoaneurysm in the rectum. A large amount of blood and massive hematoma were evacuated by surgery. The Hartmann procedure was performed, but the pseudoaneurysm was not resected. On the 11th postoperative day, hemoglobin decreased (11.6 g/dL–7.9 g/dL), and CT revealed a recurrent hematoma (6.0 × 4.2 cm) in the pelvic cavity, with a residual pseudoaneurysm. Angiography failed to localize the pseudoaneurysm. Consequently, prophylactic embolization at the anterior branch of both the internal iliac arteries was performed. The subsequent hospitalization course was uneventful.

**Discussion:**

Rectal impalement injury may result in pseudoaneurysm of the rectal arteries. However, pseudoaneurysm rupture of the mid rectal artery, followed by massive hemoperitoneum, has not been reported in the English literature. From our experience, preoperative diagnosis of a pseudoaneurysm is crucial for definite surgical management. When surgical resection is indicated, it should include the underlying pseudoaneurysm.

**Conclusion:**

Although pseudoaneurysm rupture causing hemoperitoneum after a rectal impalement injury is extremely rare, meticulous preoperative evaluation is necessary for correct management.

## Highlights

• Rectal impalement injury may cause perirectal vascular injury.
• Pseudoaneurysm formation by rectal impalement injury is rare.
• Pseudoaneurysm rupture of the mid-rectal artery followed by massive hemoperitoneum after rectal impalement injury is extremely rare.
• Preoperative radiologic evaluation is crucial for definite surgical management.
• When surgery such as involved organ resection is indicated, pseudoaneurysm, which is bleeding focus, should be included in the surgical specimen.

## 1 Introduction

Pseudoaneurysm is defined as a perivascular hematoma that maintains communication with the vascular structure, caused by partial disruption of the vascular layers. Pseudoaneurysm usually develop after penetrating trauma, stab wounds, and infection [1,2]. In case of rectal impalement injury, rectal bleeding or perforation followed by sepsis may be the main complications. However, pseudoaneurysm rupture causing massive hemoperitoneum is extremely rare. Here, we present the case of a 43-year-old man with rectal impalement injury that resulted in pseudoaneurysm rupture of the mid rectal artery, with massive hemoperitoneum. This work has been done in line with the SCARE criteria [3].

## 2 Case presentation

**Fig. 1:**
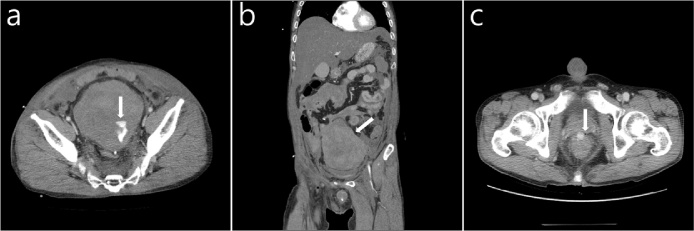
(a) Initial contrast-enhanced axial computed tomography image showing the perirectal hematoma with extravasated blood (arrow); (b) Initial contrast-enhanced coronal image showing the perirectal hematoma with hemoperitoneum; (c) Initial contrast-enhanced axial computed tomography image showing the pseudoaneurysm (arrow).

**Fig. 2:**
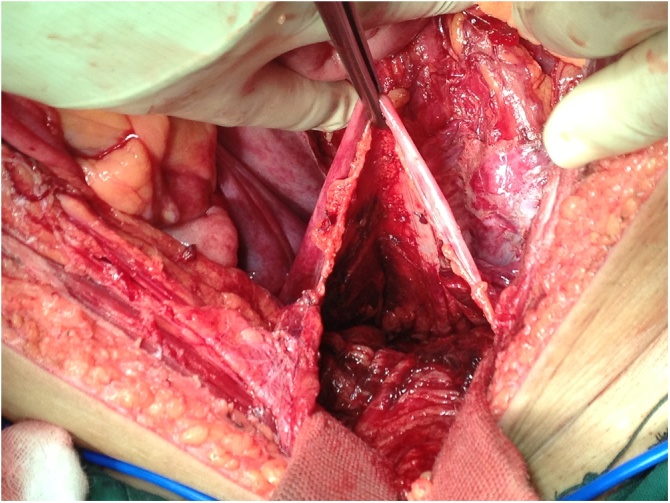
Operative findings after evacuation of the subperitoneal perirectal hematoma.

A 43-year-old man with a history of chronic alcoholism presented with abdominal distension. The previous day, the patient had presented to a local hospital with anal bleeding and abdominal pain after an incidental insertion of barbecue skewer per anus in the drunken state; subsequently, he had undergone sigmoid loop colostomy for rectal perforation. However, after the operation, the patient had become hemodynamically unstable. At presentation, his systolic blood pressure was 90 mmHg and the pulse rate was 135 beats/min. Although there was no gross rectal bleeding, the digital rectal examination revealed a penny-sized anterior rectal wall defect 6 cm from the anal verge (AV). Computed tomography (CT) revealed a hematoma (12 × 10 × 15 cm) with active bleeding in the pelvic cavity and a pseudoaneurysm in the anterior wall of the rectum (Fig. 1). Since the patient was hemodynamically unstable, an emergency operation was performed. During the operation, a massive subperitoneal hematoma in the rectovesical pouch and large amount of blood in the peritoneal cavity were found. After evacuation of the hematoma and blood, oozing continued in the rectovesical pouch (Fig. 2). Thus, compression with gauze was performed for 30 min until the oozing stopped. The Hartmann procedure was performed with the suspected bleeding focus included, but the perforation site was not included.

**Fig. 3:**
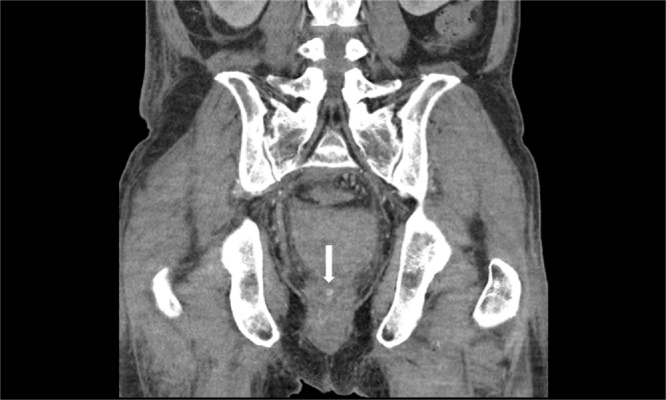
Follow-up contrast-enhanced coronal computed tomography image showing the residual pseudoaneurysm in the rectum (arrow).

**Fig. 4:**
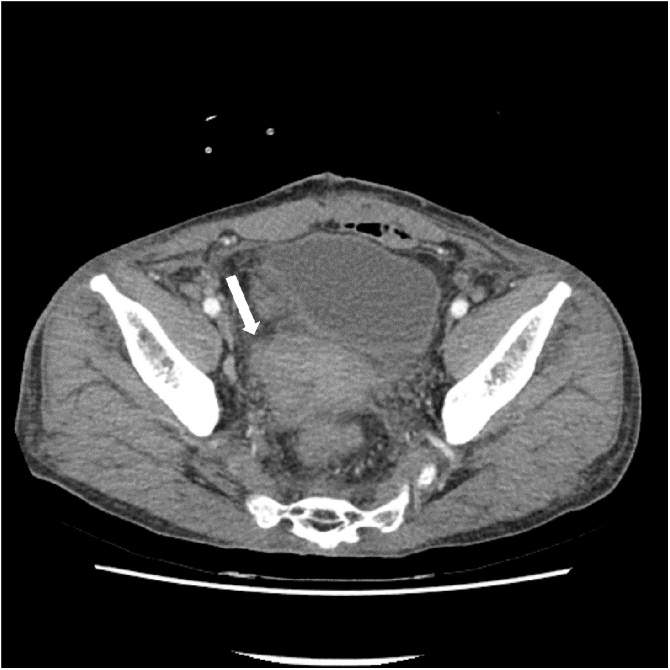
Follow-up contrast-enhanced axial computed tomography image showing the massive hematoma in the perirectal space, without any definite signs of extravasation.

**Fig. 5:**
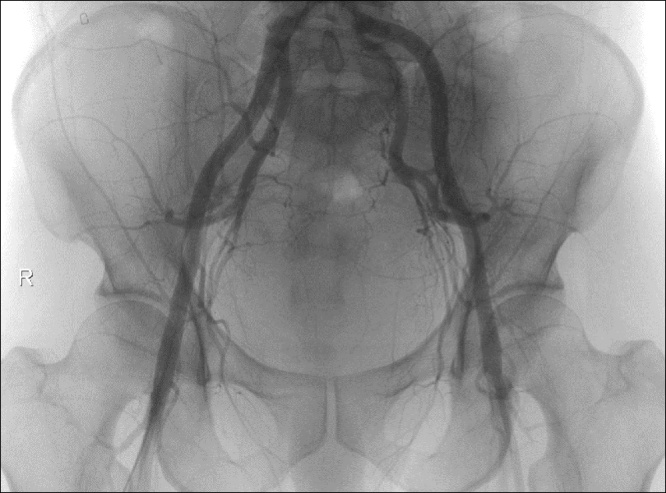
Arteriogram of the pelvis including both the internal iliac arteries, without any definite signs of extravasation.

Although the postoperative course was uneventful and there was no evidence of recurrent bleeding on the follow-up CT on the 7th postoperative day (POD), a focal enhancing lesion in the anterior wall of the rectum indicating a residual pseudoaneurysm was noted (Fig. 3). On the 11th day POD, his hemoglobin decreased from 11.6 g/dL to 7.9 g/dL, and the follow-up CT revealed recurrent hematoma (6.0 × 4.2 cm) in the pelvic cavity and the residual pseudoaneurysm (Fig. 4). Following the diagnosis of recurrent bleeding from the residual pseudoaneurysm, an angiography was performed. However, the angiography failed to localize the pseudoaneurysm, and definite signs of extravasation could not be ascertained. Thus, prophylactic gelfoam embolization at the anterior branch of both the internal iliac arteries was performed (Fig. 5). The subsequent hospital course was uneventful, and the patient was discharged on the 25th POD. After 3 months, the previous rectal lesion (AV: 6 cm) healed, and colostomy reversal was performed without morbidity.

## 3 Discussion

Rectal impalement injury may present with hematochezia and pelvic sepsis due to colorectal perforation. However, even though the vascular anatomy around the rectum is complex and the blood vessels are vulnerable to impalement injury, pseudoaneurysm formation leading to massive hemoperitoneum is extremely rare [4].

Two cases of traumatic pseudoaneurysm of the superior rectal artery have been reported, that resulted from fall down injury and colonoscopic polypectomy; however, pseudoaneurysm rupture of the mid rectal artery followed by massive hemoperitoneum has not yet been reported in the English literature [5,6].

While a true aneurysm is a localized bulge involving all the vascular layers, a pseudoaneurysm does not involve all the layers. When partial disruption of the arterial wall is not sealed, the leaked blood may dissect the adjacent tissues, forming a sac that is partially surrounded by the arterial wall layer or adjacent soft-tissue, but not by all the layers as in a true aneurysm. If the sac maintains a connection with the parent vessel, a pseudoaneurysm can ensue [1,2]. Clinical manifestations of pseudoaneurysm rupture depend on the arteries involved; based on the connected space or organ such as the bowel, the biliary system, or the peritoneal cavity, diverse clinical manifestations can develop after the rupture. Bleeding from a drain or gastrointestinal bleeding is one of the most common manifestations [[7], [8], [9]]. In case of rectal impalement injury, the symptoms related to rectal perforation such as bleeding and abdominal pain may be common [10]. In the present case, although the patient presented to the local hospital with anal bleeding and abdominal pain, the hypovolemic shock that resulted from the developing pseudoaneurysm rupture was one of the most unusual findings.

For the diagnosis of pseudoaneurysm rupture, a suspicion based on clinical manifestation is mandatory. For example, in a patient with pelvic or pancreatic surgery, sudden increase in the blood drain amount and significant reduction in the hemoglobin level requiring blood transfusion may indicate pseudoaneurysm rupture.

However, in a patient with anal impalement injury, pseudoaneurysm formation is not likely to be diagnosed in the emergency room because the presence of peritonitis is the main concern for the physician. In the present case, when we reviewed the initial preoperative CT retrospectively, the CT showed both retroperitoneal free air and the pseudoaneurysm of the mid rectal artery. However, since the patient was vitally unstable after the first operation, the diagnosis of pseudoaneurysm rupture had not been established in the emergency room. Therefore, awareness about such a rare manifestation may be helpful in the diagnosis and prompt management of a patient with rectal impalement injury.

Although conventional angiography is the standard imaging modality for the diagnosis of a pseudoaneurysm, when an interventional radiologist is not available in the emergency settings, contrast-enhanced CT may be the diagnostic modality of choice [1,11]. A contrast material-filled sac is indicative finding of a pseudoaneurysm; moreover, CT can also detect both the peritoneal and retroperitoneal lesions associated with trauma, as in the present case [10,12].

There are several therapeutic modalities for managing a pseudoaneurysm, such as angiographic embolization, stent placement, and surgery. Angiography allows an early diagnosis or prompt management in a patient with bleeding from the pseudoaneurysm; and angiographic embolization is an established treatment modality for acute bleeding [[13], [14], [15]]. However, a surgery should be performed after failure of radiologic intervention or in hemodynamically unstable patients. Pseudoaneurysm resection with bypass, ligation, or involved organ resection are surgical modalities [16]. In the present case, an emergency surgery was inevitable; however, an awareness of the mid rectal artery pseudoaneurysm preoperatively could have led to complete resection, including the causative lesion without recurrent bleeding. Thus, from our experience, while managing a patient of rectal impalement injury with unusual clinical presentation, it is crucial to collaborate with a radiologist, and pseudoaneurysm rupture may be a differential diagnosis for such a patient presenting with retroperitoneal or peritoneal hematoma after rectal impalement injury.

## 4 Conclusion

Unusual clinical manifestations may obscure the correct diagnosis of the underlying lesion, if a clinician lacks sufficient experience or knowledge. Pseudoaneurysm rupture causing hypovolemic shock after rectal impalement injury is extremely rare and its preoperative diagnosis is difficult; however, meticulous preoperative evaluation is crucial for the correct diagnosis and surgical management.

## Conflict of interest

The author has no conflicts of interest to disclose.

## Funding

This research did not receive any specific grant from funding agencies in the public, commercial, or not-for-profit sectors.

## Ethical approval

This case report has been exempted from Inje University, Ilsan Paik Hospital IRB (IRB File No. ISPAIK 2018-11-008).

## Consent

Written informed consent was obtained from the patient for the publication. A copy of the written consent is available on request.

## Author contribution

Pyongwha Choi: study concept, design, and manuscript writing.

## Registration of research studies

Research registry 4494.

## Guarantor

Pyongwha Choi.

## Provenance and peer review

Not commissioned, externally peer-reviewed.
